# 3 fraction pencil-beam scanning proton accelerated partial breast irradiation: early provider and patient reported outcomes of a novel regimen

**DOI:** 10.1186/s13014-019-1417-7

**Published:** 2019-11-21

**Authors:** Robert W. Mutter, Krishan R. Jethwa, Karthik Gonuguntla, Nicholas B. Remmes, Thomas J. Whitaker, Tina J. Hieken, Kathryn J. Ruddy, Lisa A. McGee, Kimberly S. Corbin, Sean S. Park

**Affiliations:** 1 Department of Radiation Oncology, Mayo Clinic, 200 First St SW, Rochester, MN 55905 USA; 2 Department of Surgery, Mayo Clinic, Rochester, MN USA; 3 Division of Medical Oncology, Mayo Clinic, Rochester, MN USA; 4 Department of Radiation Oncology, Mayo Clinic, Phoenix, AZ USA

## Abstract

**Background and purpose:**

To report dosimetry and early adverse effects, aesthetic, and patient-reported outcomes of a prospective study of 3-fraction pencil-beam scanning (PBS) proton accelerated partial irradiation (APBI).

**Materials and methods:**

Eligibility included women age ≥ 50 years with estrogen receptor positive (ER+), sentinel lymph node negative invasive or in-situ breast cancer measuring ≤2.5 cm. The prescription was 21.9 Gy (RBE 1.1) in 3 daily fractions to the post-operative tumor bed with a 1 cm expansion. Toxicities were collected using Common Terminology Criteria for Adverse Events (CTCAE) version 4.0, 10-point Linear Analog Scale Assessment, Patient-Reported Outcomes Version of the CTCAE, and the Harvard Breast Cosmesis Scale.

**Results:**

Seventy-six women were treated between 2015 and 2017. The median breast volume receiving 50% of prescription or more was 28%. Median mean heart, mean ipsilateral lung, and maximum skin dose were 0 Gy, 0.1 Gy, and 20.6 Gy, respectively. With a median follow-up of 12 months, no treatment-related toxicity grade ≥ 2 has been observed. Most common grade 1 adverse events were dermatitis (68%) and skin hyperpigmentation (18%). At 12 months, the only persistent toxicities were one patient with grade 1 breast edema and one patient with a grade 1 seroma. 90% of patients reported quality of life as ≥7 out of 10 (0 indicating “as bad as it can be” and 10 indicating “as good as it can be”) and 98% of patients reported excellent or good cosmesis.

**Conclusion:**

3-fraction PBS proton APBI is well tolerated with low rates of physician and patient reported early adverse effects. Follow-up is ongoing to assess late toxicities and disease control outcomes. Further investigation of this novel adjuvant treatment strategy is warranted.

## Summary

Pencil-beam scanning proton therapy is an attractive modality for accelerated partial breast irradiation (APBI) delivery due to the capacity for exceptional normal tissue sparing, including the skin. We developed and prospectively investigated a novel, 3 day ultra-accelerated fractionation regimen for the delivery of proton APBI. Low rates of early physician and patient reported adverse effects have been observed to date. Further investigation of this promising technique and dose-fractionation regimen is warranted.

## Introduction

Accelerated partial breast irradiation (APBI) is an established alternative to whole breast irradiation for selected women with biologically favorable, lymph node negative, early stage breast cancer [1, 2]. Pencil-beam scanning (PBS) proton therapy is an emerging technology that is attractive for APBI delivery. Because of sharp dose fall off at the proton Bragg Peak, proton APBI enables a highly conformal and homogeneous dose distribution, limiting exposure to the heart, lungs, and breast tissue outside of the target volume [3, 4]. In addition, PBS proton delivery in particular permits skin surface dose modulation to minimize superficial hot spots, more analogous to megavoltage photon irradiation, which may reduce the risk of acute and late skin toxicity [5–9].

The optimal dose and fractionation for proton APBI is not known. A meta-analysis of the UK Standardization of Breast Radiotherapy (START) A and START pilot trials estimated that the breast cancer α/β ratio for locoregional control is comparable to or lower than common late adverse events of breast cancer radiotherapy [10]. These results suggested that delivery of small radiotherapy fractions over a prolonged period of time may not improve the therapeutic ratio compared with treatment delivery in a more condensed manner. The most commonly employed photon external beam APBI regimen in North America has been 10, twice daily fractions of 3.85 Gy [11]. This is also the dose and fractionation used for external beam APBI delivery in the investigational arm of the randomized National Surgical Adjuvant Breast and Bowel Project (NSABP) B-39/Radiation Therapy Oncology Group (RTOG) 0413 trial comparing APBI and whole breast radiotherapy. However, this regimen has already been associated with worse cosmesis and more overall adverse effects compared with whole breast irradiation in a separate randomized study, supporting the investigation of alternative APBI schedules [8]. Of note, adverse aesthetic outcomes and toxicity following external beam APBI has been correlated with the volume of breast irradiated [12].

Given these data demonstrating the fraction size sensitivity of breast cancer relative to surrounding tissues from the START trials and the knowledge of proton therapy’s capacity to reduce irradiated breast volume, we designed and investigated a novel 3 fraction PBS APBI regimen. We further surmised that safely shortening the proton APBI schedule could be attractive across a range of health care systems to improve patient convenience and access to a promising technology that reduces exposure to healthy normal tissue.

The purpose of this manuscript is to report treatment planning and early physician-assessed adverse events and patient-reported outcomes (PROs) of 3-fraction PBS APBI.

## Materials and methods

### Patients

The study population included consecutive patients treated with 3-fraction PBS proton APBI for early stage breast cancer at the XXXX. MC1532 is a prospective, institutional board approved study evaluating daily 3-fraction intracavitary catheter-based brachytherapy, external beam 3-D conformal radiotherapy, or external beam pencil-beam scanning intensity modulated proton therapy. On this study, treatment modality was chosen according to physician and patient discretion. Eligibility criteria included women age 50 years or older with pathologic tumor size ≤2.5 cm, estrogen receptor (ER)-positive invasive breast cancer confirmed lymph node negative, or pure DCIS. Between 2015 and 2017, 51 patients (47 at XXXXXX, 4 at YYYYY) were treated with PBS proton APBI on MC1532. After enrollment to MC1532 was completed, an additional 25 patients were treated with 3-fraction PBS APBI on a prospective registry. These patients met the eligibility criteria of MC1532 and were all treated and followed according to the MC1532 protocol. Therefore, there were 76 evaluable patients included in this analysis of early physician-assessed adverse events and patient-reported health-related QoL of patients treated with 3-fraction PBS proton APBI.

The primary endpoint of MC1532 is the difference in the percentage of patients with adverse cosmesis (fair or poor cosmesis) at 3 years compared to baseline, as assessed by trained nurse providers, and will be reported for all cohorts when median follow-up is sufficiently mature.

### Treatment

The α/β for local-regional relapse was estimated at 3.5 in a meta-analysis of the START A and START pilot trials. This value was less than or equal to the estimates for breast shrinkage, breast induration, telangiectasia and breast edema [10]. Our overlying hypothesis was that the low α/β ratio for breast cancer could be exploited to safely compress treatment into a 3 fraction daily regimen. We elected a regimen of 7.3 Gy [RBE 1.1] daily for three fractions to a total dose of 21.9 Gy [RBE1.1]. Using an α/β ratio of 3.5 for both tumor control and late effects and assuming complete repair between fractions, this regimen translates into comparable 2 Gy equivalent biologically effective doses as the 15 fraction whole breast irradiation arm of START B as well as a frequently employed 3.4 Gy × 10 fraction partial breast irradiation regimen that had been associated with acceptable disease control and cosmetic outcomes to date [10, 13, 14].

Patients were immobilized on a breast board in the supine position with both arms abducted and externally rotated. A CT simulation was performed with 1-mm slices in free breathing. The clinical target volume (CTV) was defined as the tumor bed plus a 1 cm margin limited to 5 mm from the surface of the skin and excluding the chest wall and pectoralis muscles, the same expansion utilized for photon APBI on MC1532. Plans were constructed in the Eclipse (Varian Medical Systems, Palo Alto, CA) planning system using multi-field optimization which enables the use of robust optimization tools in the planning system. A median of two multi-field optimized beams were used for treatment planning. A typical beam arrangement is two beams with a 45–60 degree angle between them. We generally prefer a two-field approach over single field plans to help limit areas of high linear energy transfer, particularly near the chest wall. In some scenarios the multi-field approach may also modestly facilitate skin sparing.

Planning objectives included the following: CTV D95% ≥ 95% (95% of the target volume to receive 95% of prescription or more); ipsilateral breast V50% < 35% (< 35% of the volume, defined as all glandular breast tissue according to the RTOG atlas limited anteriorly within 5 mm from the surface of the skin, to receive 50% of prescription or more); ipsilateral breast V100% < 20% (< 20% of the volume to receive 100% of prescription or more); skin D1cc < 95% (maximum dose received by at least 1 cc of the first 3 mm beneath the body surface < 95% of prescription); chest wall D0.01cc < 100% (maximum dose received by at least 0.01 cc of the volume < 100% of prescription). We also attempt to limit the D0.01cc of the heart to less than 1 Gy. Setup uncertainty analyses of +/− 3 mm isocenter shifts in each translational axis and 3% beam range uncertainty were performed to ensure robust target coverage and normal tissue sparing (analogously, the planning target volume [PTV] expansion was 3 mm for photon APBI). Dosimetry was verified by an in-house graphic processing unit-based Monte Carlo simulation system. All doses were prescribed and are reported here in Gy relative biological effectiveness (RBE) 1.1 times the physical dose. In addition, as part of routine planning we evaluate a biologically modelled plan which assumes that RBE increases in a simple linear relation with linear energy transfer [15]. We attempt to limit the volume of tissue, in particular the chest wall, receiving 120% of prescription in this biologically modelled dose volume representation.

Patients were treated on consecutive business days. Elements of our breast treatment delivery system have previously been described [6]. All patients were treated on a Hitachi PROBEAT-V proton therapy system (Hitachi, Tokyo, Japan). In order to treat the shallow depths required, a range shifter was used with a 4.5 cm water-equivalent thickness. Patients were aligned using a kilovoltage 2-dimensional/3-dimensional image registration system and a robotic couch allowing for 6 degrees-of-freedom positioning for each fraction. After initial alignment to the ipsilateral anterior ribs and sternum, the lumpectomy cavity surgical clips were matched within 2 mm. Optical surface imaging with a 3-camera Align RT (VisionRT, London, United Kingdom) system was used to verify surface positioning after matching to clips and to monitor intrafraction motion. In cases where clips were not available for matching (9 of 76 patients), the primary match was to the anterior ribs immediately beneath the CTV followed by optical surface imaging to verify surface anatomy at beam entrance is within 3 mm tolerance from simulation. On rare occasions where clip or bony match result in optical surface imaging position > 3 mm from simulation, a CT verification scan is performed and the treatment plan is cast onto this scan to confirm robust target coverage and normal tissue sparing. Of note, PBS proton APBI treatment plans utilizing the techniques described here are highly robust. Even with setup uncertainty analyses of 7 mm isocenter shifts in each translational axis the CTV V90% under the worst case scenario is > 90% (90% of the CTV receives 90% of prescription or more, Additional file 1: Figure S1).

### Outcomes

Target coverage and normal tissue dosimetric parameters for the CTV, heart, lungs, ipsilateral breast and skin were reported as median and interquartile range (IQR). Provider assessment of early and late adverse events was performed using Common Toxicity Criteria for Adverse Events (CTCAE) version 4.0. The Patient-Reported Outcomes Version of the CTCAE (CTCAE-PRO) was used to assess patient-reported skin toxicity and 10-point Linear Analog Scale-Assessment (LASA) were utilized to assess patient-reported overall quality of life, pain, and fatigue [16, 17]. In addition, a modified Harvard Breast Cosmesis Scale was used to assess patient-reported cosmesis [18].

## Results

Between December 2015 and November 2017, 76 women underwent wide local excision with negative margins and received 3 fraction PBS proton APBI. The median time from surgery to the initiation of radiotherapy was 44 days (range 24 to 78 days). 61 (80%) had invasive breast cancer and 15 (20%) had DCIS. The estrogen receptor (ER) was positive in all cases of invasive breast cancer and 13 of 15 (87%) of DCIS. Sixty-seven percent of tumors were > 1 cm and 59% were grade 2 or 3. Patient and tumor characteristics are shown in Table 1.

**Table 1:** Patient Characteristics

Variable	*N* = 76
Age, median (range)	67 (51–81)
Laterality	
Right	41 (54%)
Left	35 (46%)
Histology	
Ductal	72 (96%)
Lobular	1 (1%)
Other	3 (3%)
Invasive	61 (80%)
DCIS	15 (20%)
Grade	
1	31 (40%)
2	39 (51%)
3	6 (8%)
Tumor size	
≤ 1 cm	25 (33%)
> 1–2 cm	47 (62%)
> 2–2.5 cm	4 (5%)
ER status	
Positive	74 (97%)
Negative	2 (3%)
Her-2 Positive	2 (3%)
Adjuvant Endocrine therapy	51 (67%)
Adjuvant Chemotherapy	1 (1%)

**Fig. 1:**
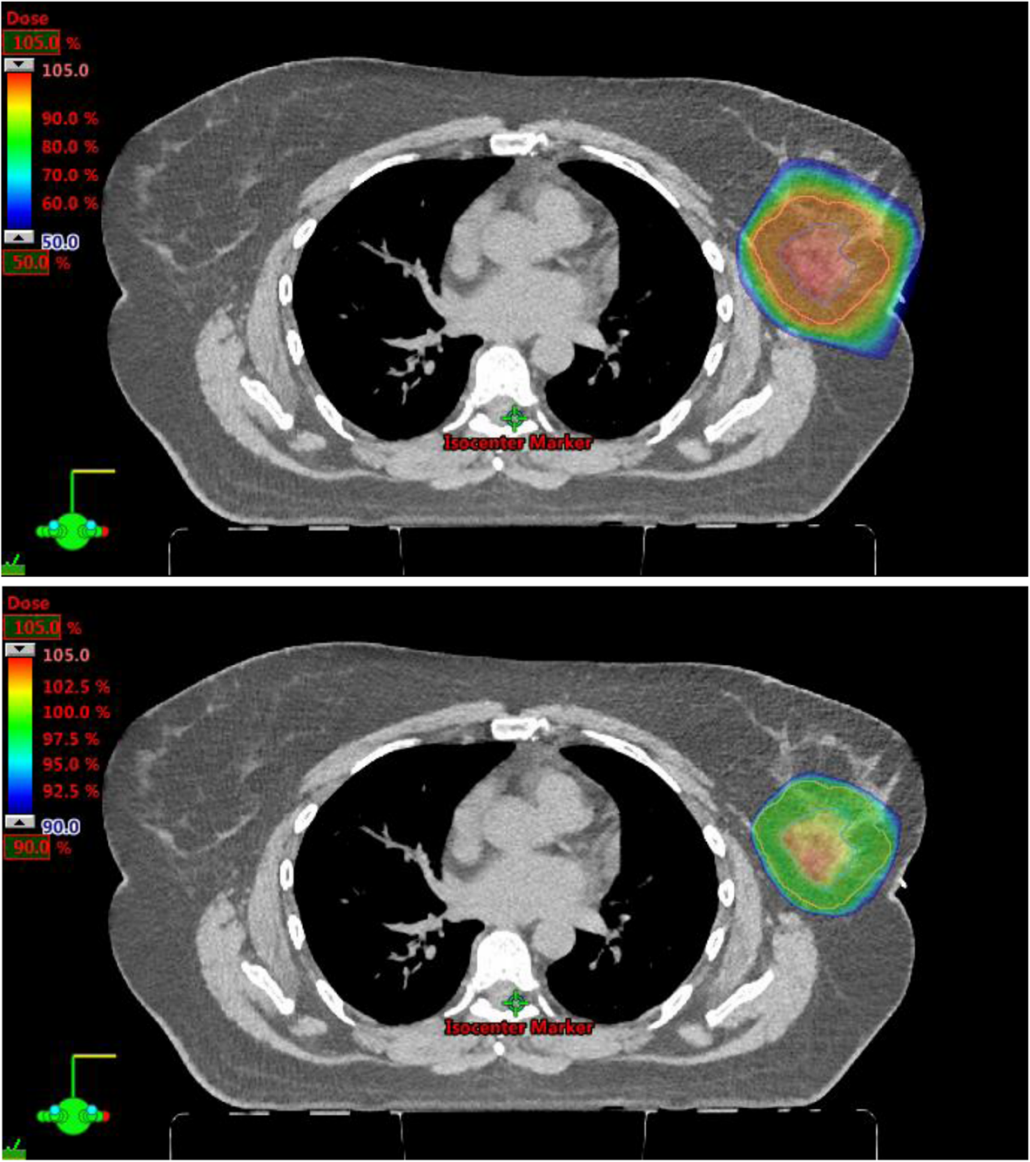
Axial CT slice of a PBS proton APBI treatment plan with 50–105% prescription color wash (above) and 90–105% prescription color wash (below) demonstrating excellent target coverage and skin, lung, heart, and other normal tissue sparing

Target coverage and doses to organs at risk are shown in Table 2. The median ipsilateral breast V50% and V100% were 27.8 and 8.4%, respectively. The median mean heart dose and median heart D0.01 cc were 0 Gy and 0.1 Gy, respectively, and the median mean ipsilateral lung dose, median ipsilateral lung V5 Gy, and median ipsilateral lung V10 Gy were 0.15 Gy, 0.4, and 0%, respectively. The median D0.01cc for the skin was 20.6 Gy, or 94.1% of prescription. Axial slices of a typical treatment plan for a patient on study demonstrating normal breast and skin sparing are shown in Fig. 1.

**Table 2:** Target and normal tissue dosimetry

Target/Organ at Risk	DVH Parameter	Dosimetry Achieved Median (IQR)
*CTV*	V95% (%)	99.1% (97.6, 99.9)
*Heart*	D0.01 cc (Gy)	0.1 Gy (0.0, 0.9)
	Mean (Gy)	0.0 Gy (0.0, 0.0)
*Lung, Ipsilateral*	Mean (Gy)	0.15 Gy (0.05, 0.31)
	V5 Gy (%)	0.4% (0.0, 1.4)
*Ipsilateral Breast*	V50% (%)	27.8% (20.7, 34.4)
	V100% (%)	8.4% (5.2–11.3)
*Skin*	D0.01 cc (Gy)	20.6 Gy (19.6, 21.1)
	D1cc (Gy)	19.5 (18.6, 20.2)

CTV, clinical target volume; V95%, the volume receiving 95% of the prescribed dose or more; D0.01cc, maximum dose received by at least 0.01 cc of the volume; V5 Gy, the volume receiving 5 Gy or more; D1cc, maximum dose received by at least 1 cc of the volume; V50%, the volume receiving 50% or more of the prescribed dose; V100%, the volume receiving 100% or more of the prescribed dose

Table 3 displays provider assessed adverse events at baseline, end of treatment, 3 months and 12 months. With a median follow-up of 12 months, there have been no grade ≥ 2 toxicities. Therefore, only grade 1 toxicity rates are displayed. The most common adverse event was grade 1 radiation dermatitis noted in 68% of patients at the end of treatment, 12% of patients at 3 months, and 0% at 12 months. 12% of patients had grade 1 skin hyperpigmentation on the last day of treatment and 18% at 3 months, but there were no reports of skin hyperpigmentation at 12 months. Grade 1 breast edema was noted in 22% of patients at the end of treatment, but just 8 and 2% at 3 months and 12 months, respectively. Besides the one patient with grade 1 breast edema the only other persistent toxicity documented at 12 months was grade 1 seroma in 1 (2%) patient.

**Table 3:** Provider-assessed adverse events

Provider-Assessed AE	CTCAE Grade	Baseline (n = 76)	Post-RT (n = 76)	3 month (*n* = 72)	12 month (*n* = 51)
*Dermatitis*	1	0%	68%	12%	0%
*Skin Hyper-pigmentation*	1	3%	12%	18%	0%
*Telangiectasia*	1	0%	0%	1%	0%
*Superficial Fibrosis*	1	0%	1%	0%	0%
*Deep fibrosis*	1	0%	1%	0%	0%
*Seroma*	1	29%	33%	11%	2%
*Chest pain*	1	0%	5%	4%	0%
*Breast Edema*	1	1%	22%	8%	2%
*Pneumonitis*	Any	0%	0%	0%	0%
*Breast Infection*	Any	0%	0%	0%	0%

**Fig. 2:**
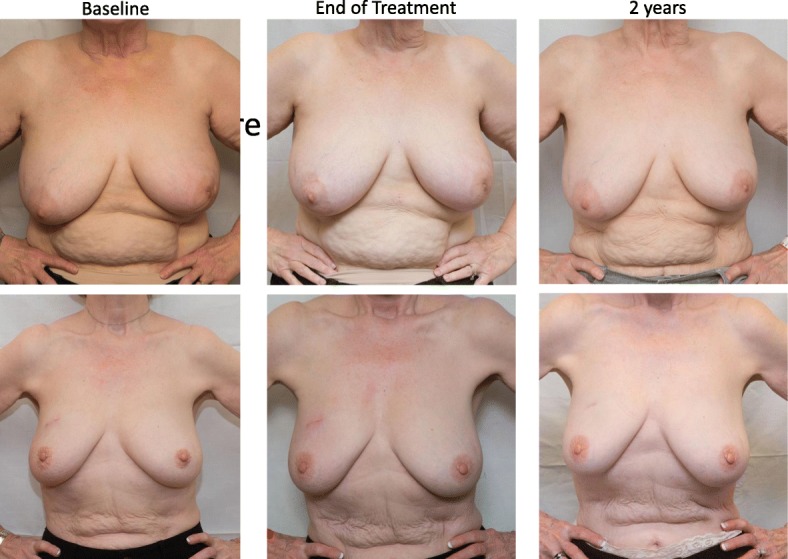
Baseline, end of treatment, and 2-year follow-up pictures of two patients treated with 3 fraction PBS proton APBI

Table 4 displays patient reported outcomes for all patients. Patient reported outcomes stratified by receipt of endocrine therapy are also provided as Additional file 2: Tables S1 and S2. At baseline, end of treatment, 3 months, and 12 months follow-up 92, 84, 78, and 80% of evaluable patients, respectively, completed the patient reported outcomes survey. Patient reported quality of life was excellent. 92, 93, and 93% of patients reporting quality of life as at least 7 out of 10 with 10 indicating quality of life “as good as it can be” and 0 indicating quality of life “as bad as it can be”, at end of treatment, 3 months, and 12 months, respectively, compared with 90% at baseline. Patient reported fatigue and pain also did not appear to be detrimentally impacted by treatment (Table 4). Mild or moderate skin toxicity at the end of treatment was reported in 23 and 9% of patients, respectively. These rates decreased to 14 and 2% at 3 months, and 10 and 5% at 12 months, respectively. Ninety-eight percent of patients reported excellent or good cosmesis at 12 months. Representative baseline, end of treatment, and 2-year photos are shown for two patients in Fig. 2.

**Table 4:** Patient-reported outcomes

Patient-Reported Outcomes	LASA^α, β^ HBCS^€^ CTCAE-PRO^‡^	Baseline (*n* = 70)	Post-RT (*n* = 64)	3 month (*n* = 56)	12 month (*n* = 41)
*Quality of Life* ^**α**^	7–10	90%	92%	93%	93%
	4–6	8%	6%	3%	7%
	1–3	2%	2%	3%	–
*Skin Toxicity*^**‡**^	None	100%	75%	86%	90%
	Mild	0%	23%	14%	10%
	Moderate	0%	9%	2%	5%
	Severe/very severe	0%	0%	0%	0%
*Pain* ^**β**^	1–3	86%	91%	84%	88%
	4–6	13%	6%	16%	10%
	7–10	1%	3%	0%	2%
*Fatigue* ^**β**^	1–3	79%	73%	73%	73%
	4–6	13%	14%	16%	20%
	7–10	9%	13%	11%	7%
*Breast Cosmesis*^**€**^	Excellent	43%	63%	62%	71%
	Good	50%	27%	36%	27%
	Fair	5%	9%	2%	2%
	Poor	2%	2%	0%	0%

**LASA**^**α**^**:** Reported on a scale of 1–10 (0 indicating “as bad as it can be” and 10 indicating “as good as it can be”)

**CTCAE-PRO**^**‡**^**:** Reported on a 5-point scale (none, mild, moderate, severe, very severe)

**LASA**^**β**^**:** Reported on a scale of 1–10 (0 indicating “none” and 10 indicating “as bad as it can be”)

**HBCS**^**€**^**:** Reported on a 4 point scale (excellent, good, fair, poor)

## Discussion

We developed and investigated a novel, ultra-accelerated fractionation regimen for the delivery of proton APBI that enables patients to complete adjuvant radiotherapy in just three days with an extremely low rate of early adverse events. Three-day PBS proton APBI was associated with impressive sparing of healthy breast tissue outside of the clinical target volume. Moreover, there was minimal lung exposure with this technique and the mean heart dose was 0 Gy. With PBS proton therapy, a magnet is used to steer the proton beam in order to paint proton spots across the treatment volume in successive layers. Therefore, this proton therapy technology has an added advantage over aperture and compensator based passive scattering proton therapy techniques in enabling modulation of the dose near the skin surface in order to reduce hot spots [5, 6]. The skin-sparing capacity of the PBS proton APBI used in our study may have contributed in part to the limited patient and provider reported dermatological adverse effects and favorable cosmetic outcomes observed thus far.

Prior proton APBI studies investigating more protracted fractionation schedules have used passive scattering techniques. With passive scattering proton therapy, material is placed in the proton beam path in order to broaden the beam and custom-made collimators and compensators are used to shape the beam to the target volume. Compared with PBS proton APBI, skin doses with passive scattering proton therapy are higher, although multiple passively scattered beams may be used to spread the entrance dose across a larger surface at the cost of increased low dose exposure to normal breast tissue [5].

Bush and colleagues previously published 5-year treatment outcomes of 100 patients that underwent passively scattered proton APBI with delivery in the prone position [19]. In that study, the 10 fractions of 4 Gy (RBE1.1) were administered over two weeks with at least 2 proton fields. The 5-year ipsilateral breast tumor recurrence-free survival was 97%. Grade 1–2 dermatitis was observed in 62% of patients and 7% of patients developed grade 1 telangiectasia. Ninety percent of patients reported a good or excellent cosmetic result. Galland-Girodet and colleagues have described long-term outcomes of a non-randomized prospective trial using either 3-dimensional conformal photon-based APBI or passively scattered proton therapy where the dose administered was 32 Gy (RBE1.1) in 8 fractions administered twice daily [9]. The seven-year incidence of local failure was 6% for the entire population. Telangiectasia, skin hyperpigmentation and adverse cosmesis were more common amongst the 19 patients treated with proton APBI although no other significant differences in late noncutaneous toxicity between patients treated with photon or proton APBI were observed. Of note, although the majority of proton patients were treated with two beam plans, only one of the fields was treated per day in the Galland-Girodet study. Delivery of a single field per day would be expected to enhance the biologically effective dose at the skin surface. In addition, 6 h between twice daily fractions may have been insufficient time for complete repair of normal tissues at the dose administered [20, 21]. Both factors likely contributed to the high rates of dermatologic toxicity and adverse aesthetic outcomes [22]. Chang et al. reported outcomes of 30 patients treated with single or two-field passively scattered proton APBI delivered in 5 daily fractions of 6 Gy (RBE1.1) [23]. With a median follow-up of 59 months, there were no ipsilateral local or regional recurrences. 69% of patients had a good or excellent cosmetic results based on physician assessment. Grade 2 skin hyperpigmentation was observed in 15 and 9% of patients at one year and three years, respectively. In follow-up there was greater deformation of the treated breast compared with the untreated breast, as assessed by the percentage breast retraction assessment (pBRA). However, the pBRA score was not significantly different amongst the 15 patients treated with two-fields. Other adverse effects included rib fractures which occurred in two patients at 6 months and two years, respectively [23].

In our study, we have not observed rib fractures or other grade 2 or greater adverse effect with a median follow-up of one year. A fixed RBE of 1.1 has historically been used in the clinic for proton planning. However, it is now recognized that the RBE rises near the end of the proton track where the ionizing density increases at the Bragg Peak and distal fall-off [24, 25]. Of note, as part of our routine treatment planning process we constrained the physical dose to the chest wall. In addition, attention was made to limiting “biological hot spots” on a biological dose distribution that uses a simple linear relationship to convert dose-averaged LET to biological dose [15]. Moreover, daily rather than twice daily fractionation was elected in order to ensure more complete recovery or normal tissues between fractions [20].

Although our study is strengthened by the inclusion of patient and provider assessments of toxicity, a limitation is the relatively short follow-up. Compliance with patient reported outcomes surveys was incomplete, a well-recognized challenge of outcomes research, but compared favorably with the literature [26]. Late responding tissues have long been recognized to be more sensitive to larger fraction sizes. That said, carefully designed regimens of 5 fractions or less have been safely employed in the palliative and definitive treatment settings across multiple malignancies, including breast cancer, with low rates of late toxicity [27–29]. Nevertheless, further investigation over a more prolonged period of time is needed to confirm whether the planning techniques we described and the regimen of 3, once daily fractions of 7.3 Gy (RBE1.1) will limit the risk of rib fractures and other late adverse effects and result in favorable long-term aesthetic outcomes [23]. Of note, Jagsi and colleagues have previously shown that the ipsilateral breast V50% and V100% are significantly correlated with cosmetic outcome following intensity-modulated photon APBI [12]. Promisingly, the mean V50% and V100% were just 27.8 and 8.4% in our study, compared with 47.9 and 27.2%, respectively, in the Jagsi study.

The annual risk of recurrence in ER-positive breast cancer remains steady out to 20 years of follow-up and no subset of favorable invasive breast cancer or DCIS has yet to be identified that does not derive a significant locoregional control benefit after wide local excision from adjuvant radiotherapy [30–33]. That said, some patients with biologically favorable disease in our study may also have reasonably been managed without adjuvant radiotherapy [32, 33]. Longer follow-up is needed to estimate the impact of 3 day proton APBI on locoregional control.

Concern regarding the cost- effectiveness of proton therapy given the higher capital and operational costs of proton therapy facilities has been the primary limiting factor to the investigation of this technology for breast cancer [34, 35]. However, using Standard Medicare Payments, Ovalle and colleagues have previously shown that the cost of even 10 fractions of proton APBI is comparable with multiple other established whole breast and partial breast adjuvant radiotherapy techniques and regimens for early stage breast cancer [36]. Number of fractions administered is an important driver of radiotherapy cost. Therefore, the cost of 3-fraction proton APBI is substantially less than 10 fraction proton APBI and would be expected to compare even more favorably with these other treatment approaches. Furthermore, even if fraction number is normalized across treatment modalities, the absolute difference in cost of delivery between treatment modalities goes down with reductions in fraction number [37]. The normal tissue sparing of 3-fraction PBS proton APBI reported here is demonstrably more favorable than prior reports of photon APBI in which doses to organs at risk were significantly correlated with late toxicity [12, 38]. Therefore, if 3 day proton APBI proves to be safe and efficacious long-term, this could be an advance for patients, payers, and health care systems alike. Further comparative effectiveness research is needed to determine the optimal patient selection for proton APBI and other APBI treatment modalities.

In summary, 3 day PBS proton APBI is feasible and associated with excellent normal tissue sparing and low rates of physician and patient reported adverse effects in early follow-up. Longer follow-up is needed to assess disease control, late toxicity and aesthetic outcomes with this regimen. However, these promising early outcomes suggest that further investigation of this ultra-accelerated regimen with both PBS and passively scattered proton therapy techniques is warranted.

## Supplementary information

**Additional file 1: Figure S1.** Axial CT slice of a PBS proton APBI treatment plan with 90–105% prescription color wash (above) and dose volume histogram (below) demonstrating CTV coverage on the base plan (solid) as well as setup uncertainty analyses of +/− 7 mm isocenter shifts in each translational axis and 3% beam range uncertainty. The worst case setup uncertainty analysis (Z:-7 mm) is highlighted demonstrating clinically acceptable target coverage of approximately 94% of the CTV receiving 95% of prescription.

**Additional file 2: Table S1.** Patient reported outcomes(PRO) in patients treated with endocrine therapy. **Table S2.** Patient reported outcomes(PRO) in patients that did not receive adjuvant endocrine therapy.

## Data Availability

All data and materials is available.
