# Supplementary figures and images for: 3 fraction pencil-beam scanning proton accelerated partial breast irradiation: early provider and patient reported outcomes of a novel regimen
Source: Radiat Oncol. 2019 Nov 21;14:211. doi: 10.1186/s13014-019-1417-7 (PMC6873533; doi:10.1186/s13014-019-1417-7)

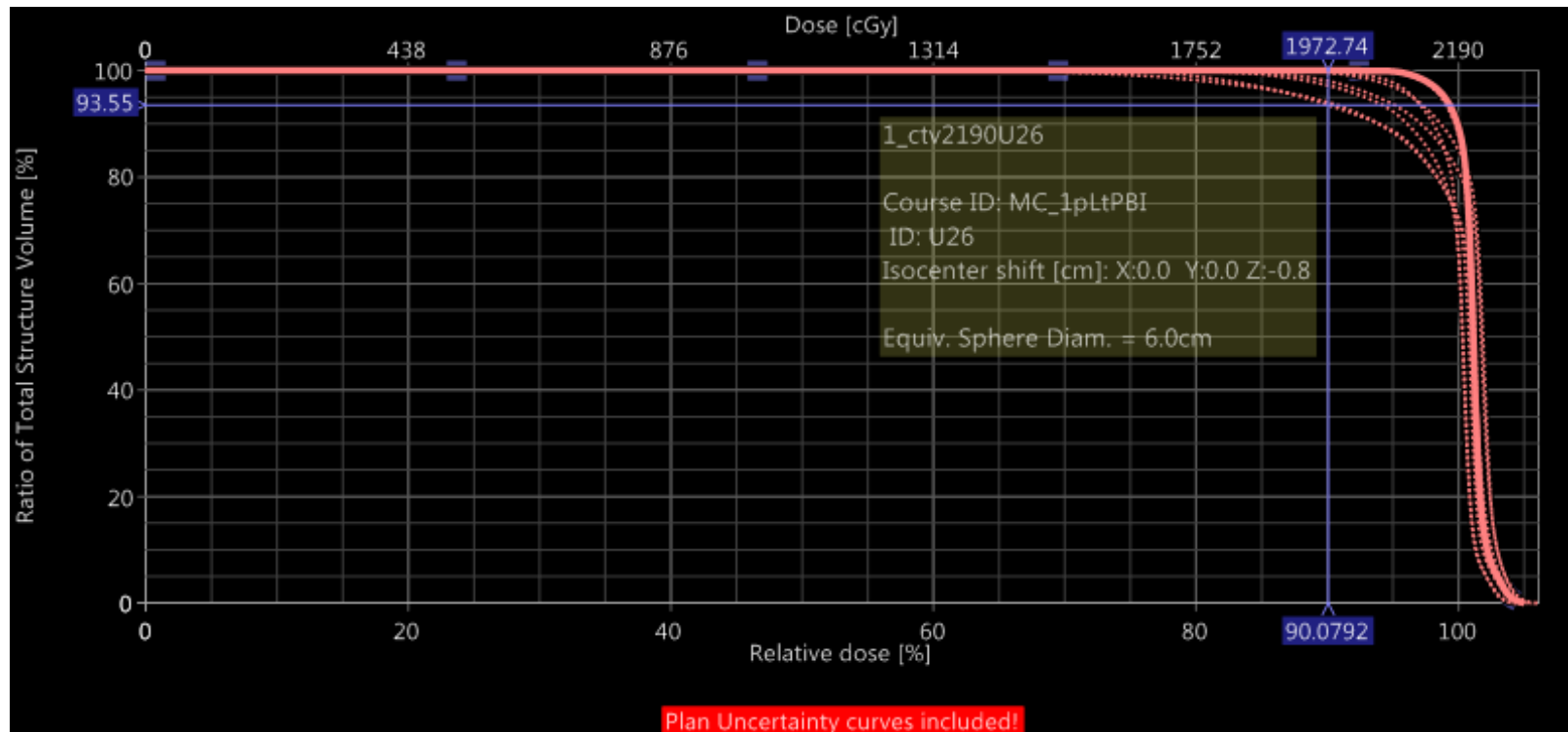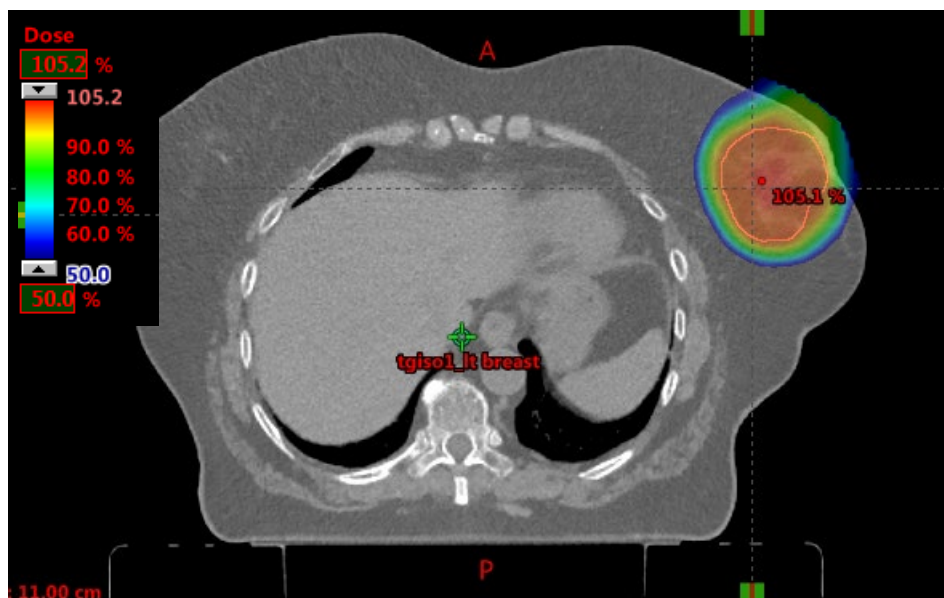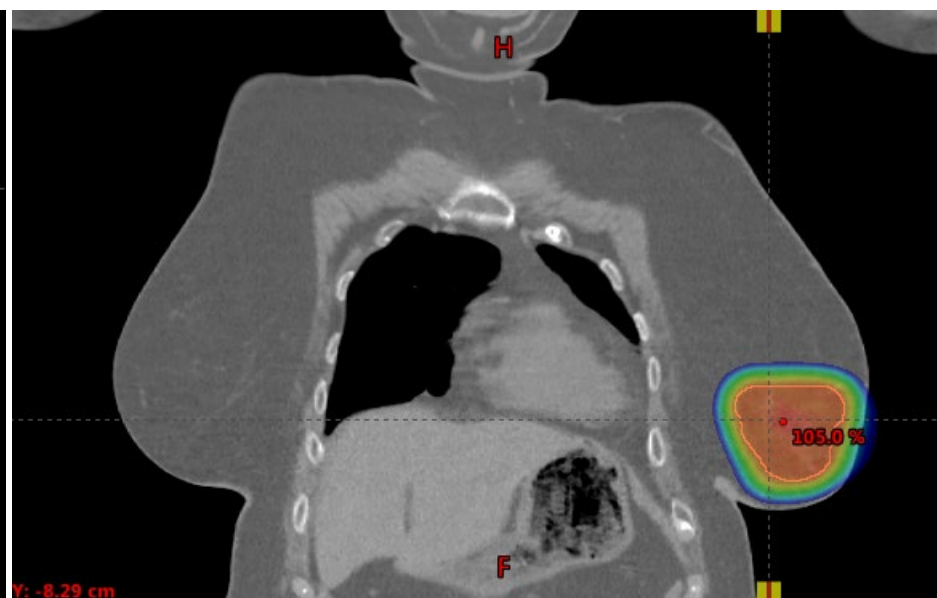

Supplement: Supplementary file 1 — Additional file 1: Figure S1. Axial CT slice of a PBS proton APBI treatment plan with 90–105% prescription color wash (above) and dose volume histogram (below) demonstrating CTV coverage on the base plan (solid) as well as setup uncertainty analyses of +/− 7 mm isocenter shifts in each translational axis and 3% beam range uncertainty. The worst case setup uncertainty analysis (Z:-7 mm) is highlighted demonstrating clinically acceptable target coverage of approximately 94% of the CTV receiving 95% of prescription. [file 13014_2019_1417_MOESM1_ESM.pdf]
