# Supplementary material for: 3 fraction pencil-beam scanning proton accelerated partial breast irradiation: early provider and patient reported outcomes of a novel regimen
Source: Radiat Oncol. 2019 Nov 21;14:211. doi: 10.1186/s13014-019-1417-7 (PMC6873533; doi:10.1186/s13014-019-1417-7)
Supplement: Supplementary file 2 — Additional file 2: Table S1. Patient reported outcomes(PRO) in patients treated with endocrine therapy. Table S2. Patient reported outcomes(PRO) in patients that did not receive adjuvant endocrine therapy. [file 13014_2019_1417_MOESM2_ESM.docx]

**Table S1: Patient reported outcomes( PRO) in patients treated with endocrine therapy.**

| **Patient-Reported Outcomes** | **LASA^α, β^**  **HBCS^€^**  **CTCAE-PRO^‡^** | **Baseline (n=48)** | **Post-RT (n=46)** | **3 month (n=37)** | **12 month (n=27)** |
| --- | --- | --- | --- | --- | --- |
| *Quality of Life* **^α^** | 7-10 | 90% | 89% | 94% | 93% |
|  | 4-6 | 8% | 11% | 3% | 7% |
|  | 1-3 | 2% | 0 % | 3% | - |
| *Skin Toxicity***^‡^** | None | 100% | 78% | 86% | 96% |
|  | Mild | 0% | 20% | 14% | 4% |
|  | Moderate | 0% | 2% | 0% | 0% |
|  | Severe/very severe | 0% | 0% | 0% | 0% |
| *Pain* **^β^** | 1-3 | 87.5% | 87% | 81% | 85% |
|  | 4-6 | 12.5% | 9% | 19% | 15% |
|  | 7-10 | 0 % | 4% | 0% | 0% |
| *Fatigue* **^β^** | 1-3 | 81% | 74% | 73% | 73% |
|  | 4-6 | 15% | 13% | 22% | 23% |
|  | 7-10 | 4% | 13% | 5% | 4% |
| *Breast Cosmesis***^€^** | Excellent | 41% | 59% | 64% | 74% |
|  | Good | 53% | 30% | 33% | 26% |
|  | Fair | 3% | 11% | 3% | 0% |
|  | Poor | 3% | 0% |  | 0% |

**LASA^α^:** Reported on a scale of 1-10 (0 indicating “as bad as it can be” and 10 indicating “as good as it can be”)

**CTCAE-PRO^‡^:** Reported on a 5-point scale (none, mild, moderate, severe, very severe)

**LASA^β^:** Reported on a scale of 1-10 (0 indicating “none” and 10 indicating “as bad as it can be”)

**HBCS^€^:** Reported on a 4 point scale (excellent, good, fair, poor)

**Table S2: Patient reported outcomes( PRO) in patients that did not receive adjuvant endocrine therapy.**

| **Patient-Reported Outcomes** | **LASA^α, β^**  **HBCS^€^**  **CTCAE-PRO^‡^** | **Baseline (n=22)** | **Post-RT (n=18)** | **3 month (n=19)** | **12 month (n=14)** |
| --- | --- | --- | --- | --- | --- |
| *Quality of Life* **^α^** | 7-10 | 82% | 100% | 89% | 86% |
|  | 4-6 | 18% | 0% | 11% | 4% |
|  | 1-3 | 0 % | 0% | 0 % | 0% |
| *Skin Toxicity***^‡^** | None | 100% | 67% | 89% | 86% |
|  | Mild | 0% | 33% | 11% | 14% |
|  | Moderate | 0% | 0% | 0% | 0% |
|  | Severe/very severe | 0% | 0% | 0% | 0% |
| *Pain* **^β^** | 1-3 | 82% | 100% | 89% | 93% |
|  | 4-6 | 14% | 0% | 11% | 0% |
|  | 7-10 | 4% | 0% | 0% | 7% |
| *Fatigue* **^β^** | 1-3 | 73% | 76% | 74% | 72% |
|  | 4-6 | 9% | 18% | 5% | 14% |
|  | 7-10 | 18% | 6% | 21% | 14% |
| *Breast Cosmesis***^€^** | Excellent | 50% | 72% | 58% | 64% |
|  | Good | 42% | 16% | 42% | 29% |
|  | Fair | 8% | 6% | 0% | 7% |
|  | Poor | 0% | 6% | 0% | 0% |

**LASA^α^:** Reported on a scale of 1-10 (0 indicating “as bad as it can be” and 10 indicating “as good as it can be”)

**CTCAE-PRO^‡^:** Reported on a 5-point scale (none, mild, moderate, severe, very severe)

**LASA^β^:** Reported on a scale of 1-10 (0 indicating “none” and 10 indicating “as bad as it can be”)

**HBCS^€^:** Reported on a 4 point scale (excellent, good, fair, poor)
